# Normal myocardial T1, T2 mapping and extracellular volume at 1.5 T in adult Thai population

**DOI:** 10.1002/acm2.70639

**Published:** 2026-05-26

**Authors:** Nabhat Noparatkailas, Nichanan Osataphan, Pakpoom Wongyikul, Phichayut Phinyo, Thanat Kanthawang, Pichitchai Atthakomol, Jinnawat Rattanang, Patanin Chindarungrueangkun, Arintaya Phrommintikul, Kwannapas Saengsin

**Affiliations:** ^1^ Division of Cardiology Department of Internal Medicine Faculty of Medicine Chiang Mai University Chiang Mai Thailand; ^2^ Center for Clinical Epidemiology and Clinical Statistics Department of Biomedical Informatics Faculty of Medicine Chiang Mai University Chiang Mai Thailand; ^3^ Clinical Epidemiology (BioCE) Faculty of Medicine Chiang Mai University Chiang Mai Thailand; ^4^ Department of Radiology Faculty of Medicine Chiang Mai University Chiang Mai Thailand; ^5^ Department of Orthopaedics Faculty of Medicine Chiang Mai University Chiang Mai Thailand; ^6^ The Northern Thailand Heart Center Faculty of Medicine Chiang Mai University Chiang Mai Thailand; ^7^ Division of Cardiology Department of Pediatrics Faculty of Medicine Chiang Mai University Chiang Mai Thailand

**Keywords:** 1.5‐T GE CMR, Asian population, cardiovascular magnetic resonance, parametric mapping, reference values

## Abstract

**Background:**

Parametric mapping is a pivotal technique in diagnostic and prognostic cardiovascular magnetic resonance (CMR). However, data pertinent to mapping parameters in healthy Asian populations, particularly with GE scanners, are limited.

**Purpose:**

To establish normal reference values for T1, T2, and ECV using 1.5‐T GE CMR in a healthy Thai population.

**Methods:**

Healthy volunteers undergoing orthopedic MRI with gadolinium injection were recruited. Health status was confirmed through medical history, ECG, laboratory investigations, and echocardiography. CMR scans included MOLLI for T1 mapping, MEFSE for T2 mapping, and ECV calculation. Inter‐ and intra‐observer variation were assessed.

**Results:**

Fifty‐one participants were included, 18 (35.29%) were males with a mean age of 41.49 ± 15.96 years. Mean global native T1, post‐contrast T1, T2, and ECV were 1037.19 ± 51.84 ms, 456.76 ± 43.98 ms, 49.93 ± 4.67 ms, and 27.06 ± 4.50%, respectively. No significant differences were observed between sexes or age group (*p* > 0.05). Reproducibility was classed as good to excellent (ICC ≥ 0.75)

**Conclusions:**

Reference values for T1, T2, post‐contrast T1, and ECV mapping in a healthy Thai population using a 1.5‐T GE scanner, were established. These findings provide critical reference data for cardiac MR examinations.

## 1 INTRODUCTION

Cardiac magnetic resonance imaging (CMR) is an invaluable tool for the diagnosis of a wide range of cardiac diseases. It has become the gold standard for the noninvasive assessment of ventricular volumes, function, and myocardial tissue characterization.^1^ Conventional techniques, such as late gadolinium enhancement (LGE), focus on signal intensity disparities between diseased and healthy myocardial tissue. However, these techniques are not always effective in the detection of diffuse interstitial fibrosis, which limits their ability to assess early myocardial abnormalities.^2^

To address this limitation, mapping methods, including T1 and T2 mapping, address the dependence on visual distinctions in signal intensities. These methods have gained widespread acceptance.^3^, ^4^ These mapping techniques provide direct, quantitative measurements of T1 (spin‐lattice) and T2 (spin‐spin) relaxation times, enabling standardized voxel‐by‐voxel signal quantification. Relaxation times vary across different tissues and are sensitive to pathological changes such as inflammation, ischemia, edema, and fibrosis.^5^ For example, unenhanced T1 mapping, or native T1 mapping, has been shown to predict all‐cause mortality and heart failure in patients with nonischemic cardiomyopathy,^6^, ^7^ while T2 mapping offers a fast, robust method for the detection of myocardial edema in acute inflammatory cardiomyopathies and can even monitor the healing of myocarditis.^8^ The mapping methods are key to evaluating tissue characterization. They vary across tissue types and within the same tissue under pathophysiological conditions including inflammation, ischemia, edema, and fibrosis.^2^

Despite the promising clinical potential of these mapping techniques, their routine use remains limited due to challenges in establishing consistent normal values, such as the vendor of the scanner and magnetic field strength. Various technical, acquisition, and patient‐dependent factors can influence mapping data, even in sophisticated facilities.^9^ As emphasized in SCMR recommendations, mapping values are highly dependent on acquisition conditions and therefore require locally derived reference ranges for accurate interpretation.

Although biological differences between ethnic groups appear to have a relatively modest effect on myocardial mapping values, prior studies have reported some variability across populations. For example, large cohort studies have suggested minimal differences attributable to ethnicity,^10^ whereas one Asian cohort demonstrated relatively lower native T1 values compared with other studies.^11^ These findings suggest that observed variations are likely influenced by both biological and methodological factors.

Given the limited availability of reference data in Asian populations using GE scanners, there remains a need to establish platform‐specific normal values in this setting. Therefore, this study aimed to determine normal reference values for native T1, T2 mapping, and extracellular volume (ECV) using a 1.5‐T GE scanner in a healthy Thai population, and to explore potential associations with age and sex.

## 2 METHODS

### 2.1 Study population

This was a prospective cross‐sectional study aimed at exploring reference T1, T2 mapping, and ECV values in healthy volunteers. Healthy volunteers were prospectively recruited from an orthopedic clinic between 02/03/2022 and 10/07/2024. Volunteers aged 18 years or older with a clinical indication for orthopedic MRI scans with gadolinium injections were screened. Inclusion criteria included the absence of cardiovascular symptoms and a normal baseline electrocardiogram (ECG). Exclusion criteria included a history or presence of cardiovascular disease (e.g., coronary artery disease, cardiomyopathy, myocarditis, atrial fibrillation, ventricular tachycardia), cardiac risk factors (e.g., hypertension, diabetes mellitus), chronic lung disease (e.g., chronic obstructive pulmonary disease, obstructive sleep apnea), chronic kidney disease (CKD) stage IV or V, pulmonary hypertension or pulmonary embolism, rheumatologic diseases (e.g., systemic lupus erythematosus, systemic sclerosis), pregnancy, or anemia.

### 2.2 Ethical approval process and inform consent

This study was approved by our local ethical review board. All participants were informed about the study objectives and procedures and provided written informed consent prior to recruitment. The study was conducted in accordance with the Declaration of Helsinki (2024), which sets ethical principles for research involving human subjects, including those related to anonymity and data protection.

### 2.3 Patient and public involvement statement

Participants were not involved in the design, conduct, reporting or dissemination plans of our research.

### 2.4 Study procedure

**FIGURE 1:**
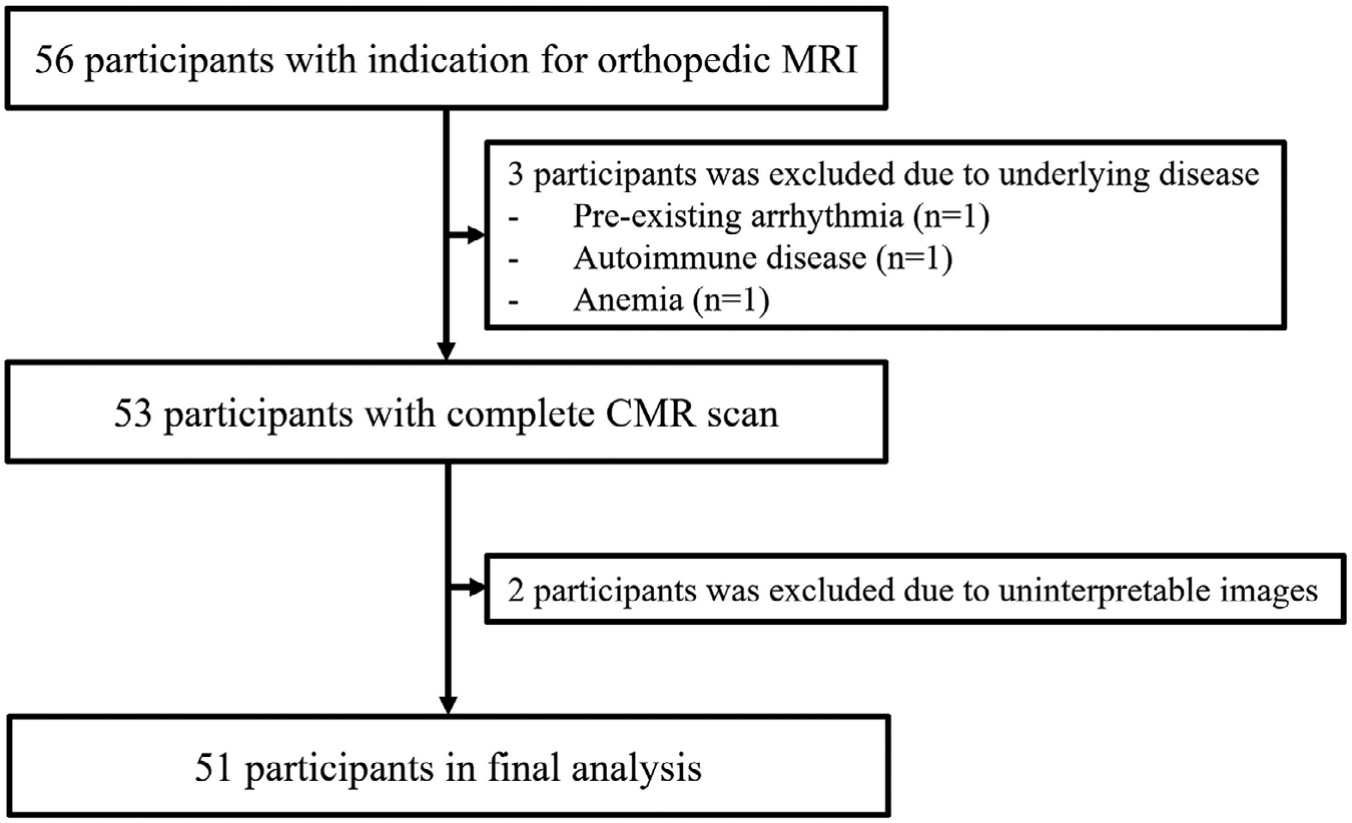
Study flow and analysis.

All volunteers underwent echocardiographic screening to exclude subclinical cardiac disease that could influence myocardial T1, T2, and ECV values. This approach, consistent with prior reference‐value studies,^11^, ^12^ was intended to ensure a truly healthy cohort rather than to pre‐define normality. Only individuals with essentially normal echocardiographic results (normal left and right ventricular systolic function, normal wall thickness, no regional wall motion abnormalities, and no more than mild valvular heart disease) proceeded to the MRI scan. During intravenous access preparation for the MRI, a venous blood sample was obtained to measure hematocrit. This was used both to exclude participants with anemia and to provide the hematocrit value required for extracellular volume (ECV) calculation. CMR with contrast was performed, followed by the orthopedic MRI protocol (Figure 1). Exposure to gadolinium during the cardiac study did not affect the soft tissue protocol. Only volunteers with normal cardiac dimensions and systolic function by cine CMR and no evidence of myocardial late gadolinium enhancement (LGE) were included in the final analysis.

#### 2.4.1 Echocardiogram protocol

A transthoracic echocardiogram was performed using a 2D imaging protocol with an IE33 Philips machine (Koninklijke Philips N.V., Netherlands) and an S5‐1 transducer. All measurements adhered to the recommendations of the American Society of Echocardiography^13^ and were analyzed by an experienced cardiologist (NO).

#### 2.4.2 MRI technique

MRI was conducted using a 1.5‐T scanner (Signa HDxt; GE Healthcare, Milwaukee, USA) with an 8‐channel phased‐array receiver surface coil. The cardiac protocols included steady‐state free precession (SSFP) cine sequences acquired with breath hold and ECG gating. Short‐axis slices (SAX) were acquired from the atrioventricular ring to the apex (8 mm thickness without gap), along with 2‐, 3‐, and 4‐chamber longitudinal views (LAX). The cine sequences were captured with 30 cardiac phases per cardiac cycle with temporal resolution ≤45 ms.

For T1 mapping, a Modified Look‐Locker Inversion Recovery (MOLLI)^14^ sequence with a 3(3s)3(3s)5 scheme was used with images acquired at three short axis slices (base, mid‐ventricle, apex) during end‐diastole with breath hold. T1 mapping was conducted pre‐ and post‐contrast (15 min after administration of 0.1 mmol/kg of Gadobutrol (Gadovist, Bayer AG, Berlin, Germany). The acquisition parameters were repetition time (TR) 3 ms, echo time (TE) 1.3 ms, flip angle 35°, spatial resolution 2 mm, and field of view 360 mm.

A multi‐echo fast spin echo (MEFSE) sequence was used for T2 mapping sequence with TR 822 ms, TE 9.6–81.7 ms (4 echoes with 24 ms increments), echo train length 20, flip angle 90°, spatial resolution 2 mm. Both MOLLI and MEFSE images were acquired at the same slice locations.

#### 2.4.3 Image analysis

The image analysis was performed by two experienced cardiologists (NN, KS) using a postprocessing software program, cvi42 (version 5.11, Circle Cardiovascular Imaging Inc., Calgary, Canada). All analyses were performed using a standardized workflow to ensure reproducibility across observers. Each interpreter independently and blinded to prior measurements. Left ventricular (LV) chamber quantification was performed by visual assessment of cine images for wall motion abnormalities. LV end‐diastolic volume (EDV), end‐systolic volume (ESV), and LV mass were determined by manual contouring of the endocardial and epicardial borders of the SAX in both systole and diastole. The absence of LGE was confirmed qualitatively through visual assessment.

**FIGURE 2:**
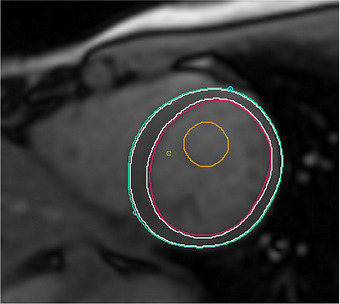
Method for region‐of‐interest (ROI) delineation on grayscale native T1 mapping images. The epicardial (green) and endocardial (red) borders were manually contoured, and a 10% offset (white) was applied to minimize partial‐volume effects. The left ventricular blood pool ROI (orange) was manually drawn, avoiding papillary muscles and endocardial borders. The mean T1 value across all segments was calculated to represent the global native T1 value.

T1 and T2 values were analyzed using scanner‐generated maps of three SAX slices located at the base, mid‐ventricle, and apex level of the LV. Following the published recommendations for myocardial mapping analysis,^5^, ^15^ the maps were displayed in grayscale for consistency. Reference points were placed at the junction of the left and right ventricle to divide the myocardium into standardized segments in accordance with the American Heart Association (AHA) guidelines. The epicardial and endocardial borders were manually contoured using software tools, and 10% offset erosion was applied to exclude the innermost and outermost 10% of myocardial thickness, thereby minimizing partial‐volume contamination while maintaining adequate myocardial sampling. This approach is consistent with previous studies.^12^ A region of interest (ROI) was placed within the LV blood pool to assess T1, T2, and extracellular volume fraction (ECV) (Figure 2). ECV was automatically calculated using the standard formula within the cvi42 software, incorporating the hematocrit value measured from a venous blood sample obtained on the same day of imaging. The mean and standard deviation (SD) of global and segmental T1, T2, and ECV values were recorded at three short‐axis levels (basal, mid‐ventricle, and apical), following SCMR consensus recommendations to represent global and regional myocardial properties.

#### 2.4.4 Statistical analysis

All statistical analyses were conducted using Stata 17 (StataCorp, Lakeway, Texas, USA). Categorical variables were summarized as frequencies and percentages, while numerical data were assessed for distribution using histograms and described as means with standard deviations (SD) or medians with interquartile ranges (IQR), depending on their distribution. Fisher's exact test was used for comparison of categorical variables, *t*‐test and Mann Whitney *U* test were used for the comparison of continuous variables as appropriate. A p‐value less than 0.05. was considered statistically significant.

Average native T1, post‐contrast T1, T2 mapping, and ECV values for each myocardial segment were visualized across age groups and sex using box and scatter plots. Linear trends were included separately for males and females, and Pearson's correlation was used to assess the strength of linear relationships.

To assess reproducibility, a subgroup of 15 participants was randomly selected for intra‐observer and inter‐observer variability using the intraclass correlation coefficient (ICC). To assess intra‐observer variability, the same investigator reassessed the myocardial tissue characteristics within one month. For inter‐observer variability, two independent cardiologists (KS and NN) performed separate analyses on the same datasets. An ICC value below 0.5 indicates poor reliability, 0.5 to 0.75 indicates moderate reliability, 0.75 to 0.9 indicates good reliability, and values above 0.90 indicate excellent reliability.^16^

## 3 RESULT

Fifty‐six volunteers were initially recruited, but five were excluded due to the presence of pre‐existing arrhythmia (*n* = 1), underlying autoimmune disease (*n* = 1), anemia (*n* = 1), and uninterpretable mapping images (*n* = 2) (Figure 1). The final analysis consisted of 51 participants, with 18 males (35.29%) and a mean age of 41.49 ± 15.96 years. The majority of participants were in the 18–40 years age group (24, 47.06%), followed by 41–60 years (17, 33.33%) and > 60 years (10, 19.61%). The mean BMI was 23.37 ± 3.55 kg/m^2^.

Out of the 51 patients, 28 (54.90%) underwent MRI of the hand and wrist, with 15 cases diagnosed as ganglion cysts. Twelve (23.53%) underwent MRI of the ankle, with 7 cases diagnosed as tenosynovitis. The remaining MRIs were of the elbow, shoulder, and forearm.

### 3.1 CMR parameters

CMR parameters are summarized in Table 1. Briefly, the mean LV end‐diastolic volume index (LVEDVi), and LV ejection fraction (LVEF) were 70.87 ± 12.19 mL/m^2^ and 62.00 ± 3.59%, respectively. The mean RV end‐diastolic volume index (RVEDVi) and RV ejection fraction (RVEF) were 75.34 ± 16.55 mL/m^2^ and 56.23 ± 5.98%, respectively. LV mass, LVEDVi, and RVEDVi were higher in men than in women, but there were no significant differences in LVEF and RVEF between both sexes.

**TABLE 1:** Participant characteristics and CMR ventricular parameters according to gender.

	Overall (Total *n* = 51)	Male (Total *n* = 18)	Female (Total *n* = 33)	*p*‐value*
Age groups, years				0.407
18‐40, *n* (%)	24 (47.1)	8 (44.4)	16 (48.5)	
40‐60, *n* (%)	17 (33.3)	8 (44.4)	9 (27.3)	
>60, *n* (%)	10 (19.6)	2 (11.2)	8 (25.2)	
BMI (kg/m^2^)	23.37 ± 3.55	23.85 ± 2.93	23.12 ± 3.86	0.489
BSA (m^2^)	1.69 ± 0.03	1.81 ± 0.17	1.63 ± 0.17	<0.001
HCT (%)	42.71 ± 3.97	44.81 ± 3.95	41.56 ± 3.54	0.004

Ventricular parameters				
LV mass (g)	80.97 ± 20.82	98.59 ± 17.12	71.36 ± 15.87	<0.001
Indexed LV mass (g/m^2^)	47.44 ± 9.03	54.34 ± 6.99	43.68 ± 7.76	<0.001
LVEDV (ml)	120.52 ± 27.51	139.10 ± 26.31	110.39 ± 22.68	<0.001
Indexed LVEDV (ml/m^2^)	70.87 ± 12.19	76.53 ± 11.07	67.78 ± 11.80	0.013
LVEF (%)	62.00 ± 3.59	62.46 ± 3.20	61.75 ± 3.81	0.506
RVEDV (ml)	128.69 ± 36.71	156.44 ± 36.71	113.55 ± 26.83	<0.001
Indexed RVEDV (ml/m^2^)	75.34 ± 16.55	85.89 ± 16.33	69.59 ± 13.77	<0.001
RVEF (%)	56.23 ± 5.98	54.46 ± 5.21	57.20 ± 6.23	0.118

Data are presented as mean ± SD.

*Male vs. female.

Abbreviations: BMI, body mass index; BSA, body surface area; HCT: hematocrit; LV, left ventricle; LVEDV, left ventricular end‐diastolic volume; LVEF, left ventricular ejection fraction; RVEDV, right ventricular end‐diastolic volume; RVEF, right ventricular ejection fraction.

**FIGURE 3:**
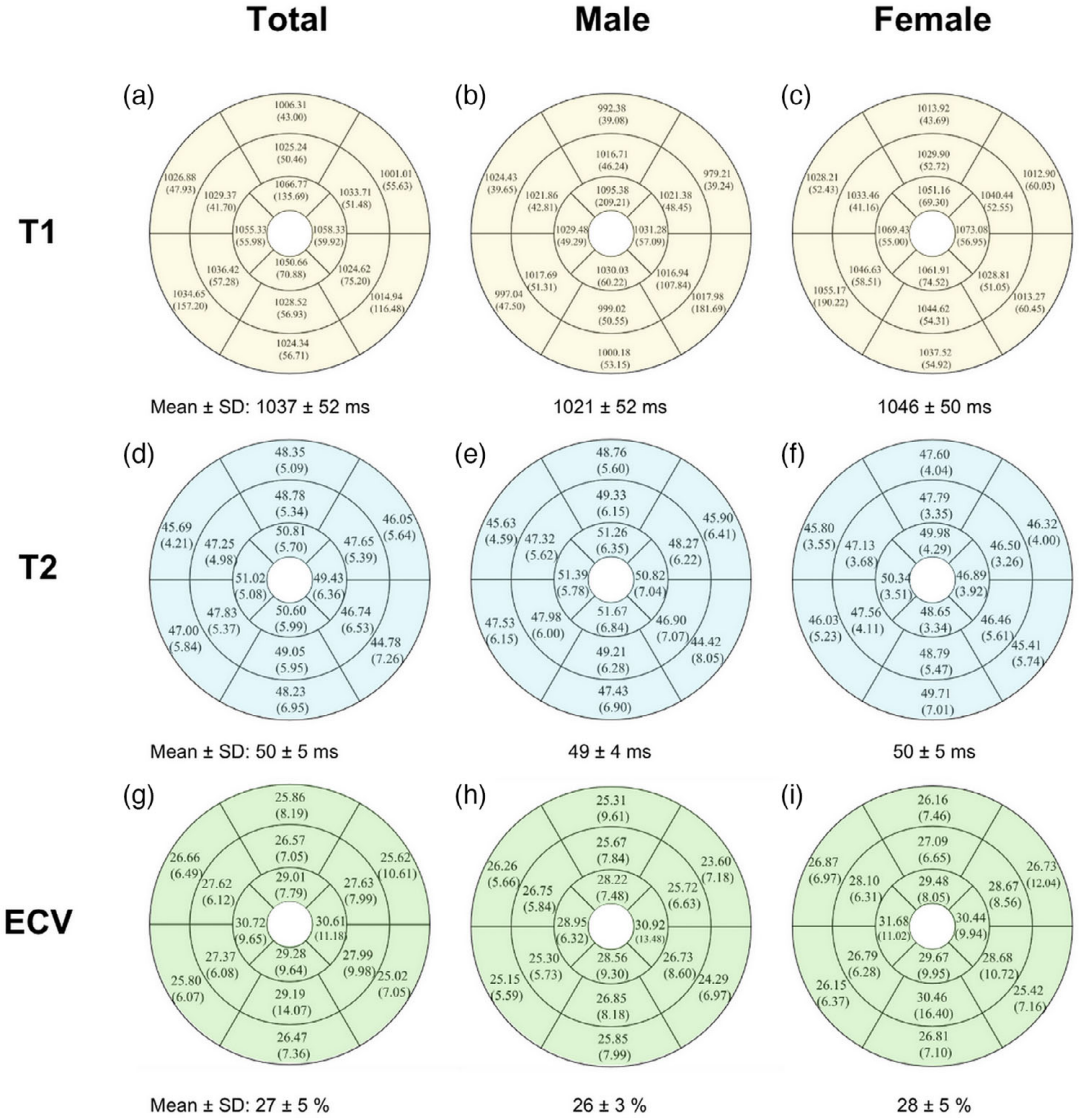
T1, T2, and extracellular volume (ECV) values displayed as bull's‐eye plots according to the American Heart Association (AHA) 16‐segment model for the overall cohort and by sex. Native T1 values are shown in panels A–C, T2 values in panels D–F, and ECV values in panels G–I. Each segment displays the mean value with standard deviation (SD). The corresponding global mean ± SD is presented beneath each bull's‐eye plot.

The mean global native T1 value was 1037.19 ± 51.84 ms, while the mean post‐contrast T1 value was 456.76 ± 43.98 ms. The mean T2 value was 49.93 ± 4.67 ms, and the mean ECV was 27.06 ± 4.50%. Reference ranges were defined according to SCMR recommendations as the mean ± 2 standard deviations and are presented in the graphical abstract. Values for T1, T2, and ECV at basal, mid‐ventricular, and apex levels are shown in Table 2. There were no significant differences in mean global native T1 mapping, T2 mapping, or ECV between sexes (Figure 3).

**TABLE 2:** Parametric mapping values based on level and average overall and by sex.

	Overall		Male		Female		
Tissue characteristic	Mean	SD	Mean	SD	Mean	SD	P‐value
T1 mapping pre‐contrast (ms)							
‐ Base	1010.43	64.53	999.94	44.08	1016.15	73.34	0.397
‐ Mid	1038.29	54.65	1009.61	36.08	1053.94	57.09	0.05
‐ Apex	1062.84	68.14	1052.83	86.49	1068.30	56.51	0.444
‐ Average	1037.19	51.84	1020.80	52.32	1046.13	50.10	0.096

T1 mapping post‐contrast (ms)							
‐ Base	469.78	76.58	502.33	117.14	452.03	30.87	0.023
‐ Mid	457.55	39.69	475.83	45.49	447.58	32.73	0.014
‐ Apex	442.96	48.03	464.78	50.74	431.06	42.70	0.015
‐ Average	456.76	43.98	480.98	52.27	443.56	32.58	0.003

T2 mapping (ms)							
‐ Base	47.38	4.70	46.40	4.36	47.90	4.86	0.278
‐ Mid	49.30	5.74	49.01	5.09	49.46	6.14	0.792
‐ Apex	53.57	4.15	52.57	4.22	54.15	4.06	0.202
‐ Average	49.93	4.67	49.33	4.11	50.26	4.98	0.500
ECV (%)							
‐ Hct	42.71	3.97	44.81	3.95	41.56	3.54	0.004
‐ Base	25.08	3.92	24.71	3.48	25.27	4.17	0.633
‐ Mid	27.30	5.68	25.59	4.02	28.18	6.25	0.128
‐ Apex	28.02	6.33	27.94	5.97	28.06	6.60	0.950
‐ Average	27.06	4.50	26.08	3.48	27.57	4.91	0.272

Abbrevation: ECV, extracellular volume fraction.

#### 3.1.1 Age and mapping value

**FIGURE 4:**
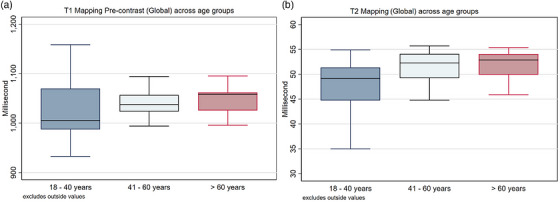
Global native T1 and T2 mapping across age groups. Boxplots showing the global native T1 (A) and T2 (B) mapping values (in milliseconds) comparing three different age groups: 18–40 years, 41–60 years, and > 60 years.

**FIGURE 5:**
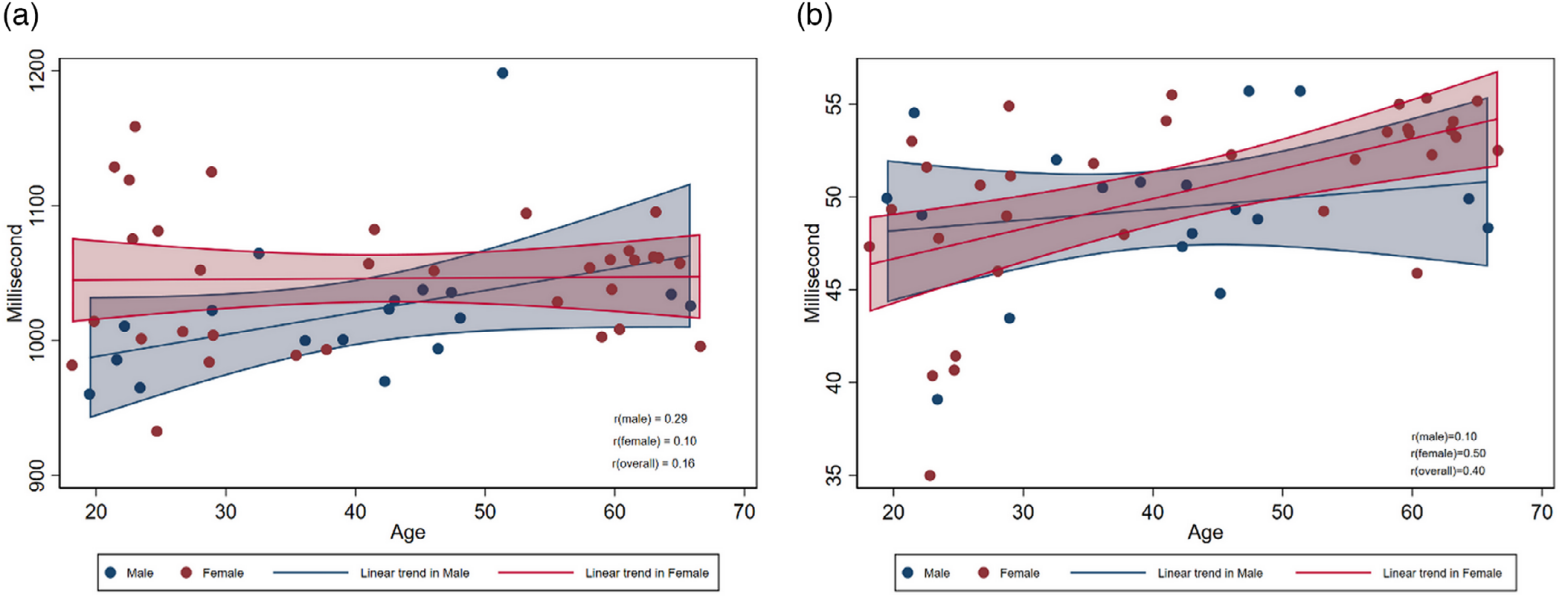
Association between age and native T1 and T2 mapping by sex. Scatter plots showing the native T1(A) and T2(B) mapping values, plotted against age for male and female subjects. Linear regression lines are added for males (blue lines) and females (red lines), with shaded regions representing the 95% confidence intervals. The correlation coefficients (r) for T1 and males, females, and the overall cohort are 0.29, 0.10, and 0.16, respectively. The correlation coefficients (r) for‐ T2 and males, females, and the overall cohort are 0.10, 0.50, and 0.40, respectively.

The global mean T1 and T2 mapping values by age group are presented in Figure 4. There were no significant differences in T1 mapping values across age groups. The correlation between T1 mapping values and age was weak (Figure 5), with correlation coefficients (r) of 0.29 for males, 0.10 for females, and 0.16 overall. Similarly, no significant differences were observed in T2 mapping values between age groups; however, the correlation between T2 mapping and age was stronger in females (*r* = 0.50) compared to males (*r* = 0.10).

#### 3.1.2 Inter‐observer agreement

Reproducibility for global native T1 mapping, post‐contrast T1 mapping, T2 mapping, and ECV scored as good to excellent (ICC ≥ 0.75) (Table 3). T2 mapping showed the highest reliability, while ECV mapping had relatively lower but still acceptable agreement.

**TABLE 3:** Inter‐observer variation of each measurement.

	ICC	95%CI	*p*‐value
Native T1 basal level	0.945	0.840 – 0.981	<0.001
Native T1 mid level	0.867	0.614 – 0.955	<0.001
Native T1 apex level	0.807	0.441 – 0.934	0.002
Post T1 basal level	0.903	0.721 – 0.967	<0.001
Post T1 mid level	0.873	0.632 – 0.957	<0.001
Post T1 apex level	0.799	0.419 – 0.932	0.002
ECV basal level	0.896	0.699 – 0.965	<0.001
ECV mid level	0.792	0.400 – 0.930	0.002
ECV apex level	0.829	0.505 – 0.942	0.001
T2 basal level	0.950	0.856 – 0.983	<0.001
T2 mid level	0.967	0.904 – 0.989	<0.001
T2 apex level	0.919	0.766 – 0.973	<0.001

Abbrevations: ECV, extracellular volume fraction; ICC, intraclass correlation.

## 4 DISCUSSION

This is the first study to provide comprehensive reference values for native T1, T2, and ECV in a healthy adult Thai population using a 1.5 GE scanner. These findings address a significant gap in CMR data for Asian populations, where normal myocardial mapping values remain underrepresented.

The global native T1 value in our study (1037.19 ± 51.84 ms) is in alignment with previously published data from 1.5 scanners using the same MOLLI technique.^3^, ^12^, ^17^ For example, a European study, conducted with a 1.5 T scanner (Signa Artist; GE Healthcare, Milwaukee, USA), recorded a T1 mapping value of 1015 ± 37 ms in participants with comparable profile.^17^ Although ethnic differences in myocardial mapping values have been reported to be relatively modest,^10^ some variability across populations has been observed. In particular, a study in an Indian cohort using a 1.5‐T Siemens scanner (Aera; Siemens, Erlangen, Germany) reported lower native T1 values (946.86 ms).^11^ These differences likely reflect a combination of biological and methodological factors; however, as emphasized in the Society for Cardiovascular Magnetic Resonance (SCMR) consensus statement,^5^ technical factors such as scanner vendor, field strength, and acquisition protocol have a greater impact on mapping values than ethnicity alone. Therefore, establishing local, platform‐specific reference values is essential for accurate clinical interpretation. Further large‐scale, multicenter studies are needed to better define the contribution of ethnicity to myocardial mapping values.

Our data demonstrated slightly higher mean native T1 values in females, consistent with prior studies using 1.5‐T scanners.^18^ Sex and age have previously been reported to influence myocardial T1 values, with some studies advocating for sex‐specific reference ranges.^19^ However, no statistically significant differences between sexes were observed in our cohort.

The mean T2 mapping value (49.93 ± 4.67 ms) was consistent with previously reported values for 1.5‐T scanners.^11^, ^20^ We observed a modest positive correlation between T2 values and age, particularly in females, which has also been described in previous studies.^21^ This finding may reflect subtle age‐related myocardial changes, such as increased tissue water content or early interstitial remodeling, although further investigation in larger cohorts is warranted.

The mean ECV value of 27.06 ± 4.50% was comparable to values reported in Western populations using different scanner platforms.^12^ ECV is well‐established marker of diffuse myocardial fibrosis and has been associated with adverse cardiovascular outcomes across a range of cardiac diseases. In our cohort, no significant differences in ECV were observed according to sex or age group, supporting the relative stability of ECV measurements in healthy individuals.

Our mean parametric mapping values were calculated from basal, mid‐ventricular, and apical slices to represent the average value of the entire left ventricular myocardium. Although a recent study^22^ reported greater variability in apical measurements due to partial‐volume effects, we intentionally included the apical slice to achieve full‐ventricular coverage. To minimize blood‐pool contamination and apical artifacts, a 10% endocardial and epicardial offset was applied during contouring. Notably, our results demonstrated comparable mapping values to previous studies and high reproducibility, as reflected by narrow standard deviations and excellent intraclass correlation coefficients across all slice levels.

The reference values reported in this study should be interpreted as platform‐specific ranges derived under standardized acquisition and analysis conditions. In clinical practice, measured parametric mapping values should be compared with these reference ranges (defined as mean ± 2 standard deviations) only when imaging parameters, scanner platform, and post‐processing methods are comparable. Values outside this range may suggest abnormal myocardial tissue characteristics and should be interpreted in the appropriate clinical context.

The strengths of our study include rigorous inclusion criteria to ensure a truly healthy cohort of participants, standardized imaging protocols, and robust interobserver reproducibility. More importantly, our study included post‐contrast parametric mapping values, the ECV, as all participants underwent scanning with gadolinium injections. This is a unique feature, as most studies reporting 1.5‐T values do not include T1, T2, and ECV in the same population.

However, several limitations must be acknowledged. First, the sample size, while sufficient for establishing preliminary reference values and consistent with SCMR recommendations for scenarios involving larger‐magnitude biological differences,^5^ remains relatively small for broader generalizability and for deriving high‐precision, age‐ and sex‐specific reference ranges. In particular, the limited number of participants in older age groups (>60 years) restricts robust subgroup analysis. Although the cohort size is comparable to prior reference‐value studies using parametric mapping, larger sample sizes are required to establish more robust and generalizable normal ranges.

Second, recruitment from an orthopedic imaging setting may introduce potential selection bias. This approach was primarily adopted for feasibility and safety, as participants were already scheduled for MRI with gadolinium contrast, allowing additional cardiac imaging without further contrast exposure. Although musculoskeletal conditions are unlikely to directly influence myocardial tissue characteristics, this hospital‐based recruitment strategy may limit the generalizability of our findings to the broader healthy population. To mitigate this limitation, all participants underwent comprehensive screening, including clinical assessment, electrocardiography, laboratory evaluation, echocardiography, and CMR, to exclude underlying cardiovascular or systemic disease. Nevertheless, future studies with community‐based recruitment and larger, more diverse populations are warranted to validate these findings.

Third, our findings are specific to the 1.5‐T GE platform and may not be directly generalizable to scanners from other vendors or field strengths. Moreover, as all participants were Thai, the results may not fully represent the diversity of all Asian populations. Nevertheless, these data provide meaningful reference values for Southeast Asian cohorts and for users of the GE 1.5‐T system, where such information remains limited.

Future studies should aim to include larger, more diverse populations across multiple centers to improve external validity. In particular, adequately powered studies are needed to derive age‐ and sex‐specific reference values with greater precision. Additionally, multicenter collaborations incorporating different vendors and field strengths would be valuable to enhance the generalizability and clinical applicability of myocardial mapping reference ranges.

## 5 CONCLUSION

In this study, we established reference values for parametric mapping, including T1, T2, post‐contrast T1, and ECV, in a healthy Thai population using a 1.5‐T GE scanner. These findings provide reference data that may support clinical and research applications on similar systems.

## AUTHOR CONTRIBUTIONS

N.N. conducted data analysis, interpreted the results, and contributed to the initial drafting of the manuscript. N.O., T.K., P.A., J.R., and P.C. were responsible for data collection. P.W. and P.P. performed data analysis and statistical analysis. A.P. and K.S. conceived and designed the study, interpreted the results, and contributed to the revision of the manuscript. All authors read and approved the final version of the manuscript.

## CONFLICT OF INTEREST STATEMENT

The authors declare no conflicts of interest.

## ETHICS STATEMENT

Ethical approval for this study was granted by the Institutional Review Board of the Faculty of Medicine, Chiang Mai University (Approval ID: PED‐2564‐08643, including any subsequent amendments). All participants provided informed consent. This study adhered to the ethical guidelines of the Declaration of Helsinki.

## DISCLOSURE OF ARTIFICIAL INTELLIGENCE (AI) PROGRAMS

This work did not use any artificial intelligence program for data analysis or manuscript writing.

## Data Availability

The data that supports the findings of this study are available from the corresponding author upon reasonable request.
